# The 1,7-malaria reactive community-based testing and response (1,7-mRCTR) approach in Tanzania: a cost-effectiveness analysis

**DOI:** 10.1186/s40249-024-01261-w

**Published:** 2024-12-04

**Authors:** Radhika Pradip Tampi, Duoquan Wang, Salim Abdulla, Muhidin Kassim Mahende, Tegemeo Gavana, Hajirani M. Msuya, Augustine Kuwawenaruwa, Michael Mihayo, Felix Brown, Honorati Masanja, Wilbald Anthony, Katia Bruxvoort, Fadhila Kihwele, Godlove Chila, Wei Chang, Marcia Castro, Xiao Ning, Prosper P. Chaki, Yeromin P. Mlacha, Jessica Cohen, Nicolas A. Menzies

**Affiliations:** 1 Program in Health Policy, Harvard University, Cambridge, MA USA; 2 National Institute of Parasitic Diseases, Chinese Center for Disease Control and Prevention (Chinese Center for Tropical Diseases Research), NHC Key Laboratory of Parasite and Vector Biology, WHO Collaborating Center for Tropical Diseases, National Center for International Research On Tropical Diseases, Shanghai, China; 3 School of Global Health, Chinese Center for Tropical Diseases Research, Shanghai Jiao Tong University School of Medicine, Shanghai, China; 4 Ifakara Health Institute, #5 Ifakara Street, Plot 463 Mikocheni, P.O. Box 78 373, Dar es Salaam, Tanzania; 5 Africa Academy for Public Health, Dar-es-Salaam, Tanzania; 6 School of Public Health, University of Alabama at Birmingham, Birmingham, AL USA; 7 World Bank Group, Washington, DC USA; 8 Department of Global Health and Population, Harvard T.H. Chan School of Public Health, Boston, MA USA

**Keywords:** Cost-effectiveness analysis, Malaria, Reactive case detection, 1,7-mRCTR, Tanzania

## Abstract

**Background:**

Reactive case detection (RACD) for malaria control has been found effective in low transmission settings, but its impact and cost-effectiveness in moderate-to-high transmission settings are unknown. We conducted an economic evaluation alongside an empirical trial of a modified RACD strategy (1,7-mRCTR) in three moderate-to-high malaria transmission districts in Tanzania.

**Methods:**

The costs and cost savings associated with the intervention relative to passive case detection alone were estimated in the study sites of Kilwa, Kibiti, and Rufiji districts in Tanzania from 2019–2021. Empirical cost data were collected using household surveys. The incremental costs of the intervention were calculated from under a societal perspective. Costs are reported in 2022 US dollars.

Trial data and malaria registers from health facilities were used to calculate the number of malaria cases detected. We simulated unobserved and distal health effects of the intervention to assess cost-effectiveness in terms of incremental cost-effectiveness ratios (ICERs).

Propagated uncertainty was assessed via second-order Monte Carlo simulation, including bootstrapping of empirical data distributions. Incremental costs per disability-adjusted life year (DALY) averted were compared to a willingness-to-pay threshold based on estimated opportunity costs of healthcare spending in Tanzania.

**Results:**

The programmatic cost of the 1,7-mRCTR intervention was 5327 United States Dollars (USD) per 1000 population. The combination of reactive and passive case detection in the intervention arm resulted in an additional 445 malaria cases detected per 1000 compared to passive detection alone, yielding an incremental cost per additional case detected of 12.0 USD. Based on modelling results, for every percentage point decline in malaria prevalence, the intervention averted 95.2 cases and 0.04 deaths per 1000 population. On average, the 1,7-mRCTR intervention averted 19.1 DALYs per 1000 population. Compared to passive malaria detection, the ICERs for the 1,7-mRCTR intervention were 7.3 USD per case averted, 16,884 USD per death averted, and 163 USD per DALY averted.

**Conclusions:**

Our analysis demonstrates that the 1,7-mRCTR intervention appears to be cost-effective under a willingness-to-pay threshold of 417 USD per DALY averted, showing that modified RACD strategies can provide value for money in moderate-to-high transmission settings.

**Graphical Abstract:**

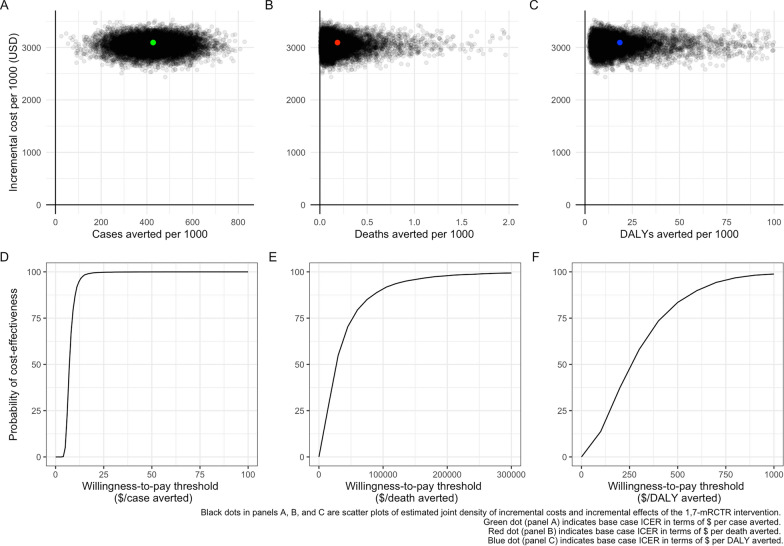

## Background

Over the past six decades, malaria control programs have been successful in reducing the incidence and deaths associated with the disease in high-burden settings [1]. While mass interventions have historically represented an important malaria control option, these intensive population-level interventions become increasingly cost-inefficient as transmission and incidence decline [2]. In many countries, malaria epidemiology is now characterized by local heterogeneity in malaria risk and transmission patterns, with optimal control strategies varying across sub-national areas. The World Health Organization (WHO) Global Malaria Programme recommends the use of surveillance data to tailor control efforts to local levels of transmission [3].

Reactive case detection (RACD) is a form of surveillance and response strategy in which a passively detected index case triggers the deployment of a local screen-and-treat response targeted at individuals living near the index case [4]. In China, the “1-3-7” strategy is a form of RACD that has been recognized as a core component of a highly successful national malaria elimination effort [5–8]. This approach requires adherence to a strict timeline; malaria cases must be reported to the Chinese Information System for Disease Control and Prevention within one day, the case confirmed and investigated by the County CDC within three days, and foci response deployed by the County CDC within seven days, giving the model its “1-3-7” name. Efforts to share the concept of the “1-3-7” RACD model in other low-endemic countries have been successful, and are now being explored in higher-endemic settings [9–11]. However, due to the resource-intensive nature of these strategies, implementation of RACD in higher-endemic settings requires adaptation to avoid undue strain on local health systems. Assessing the impact, resource utilization, and cost-effectiveness of these modified RACD strategies is critical in understanding whether these interventions provide value for money in moderate-to-high malaria transmission settings.

Tanzania has one of the highest malaria burdens in the world, but transmission intensity varies significantly across regions [12]. Improvements in surveillance and health information systems have allowed the Tanzania Ministry of Health to explore the efficiency and effectiveness of locally tailored surveillance and response approaches across regions with varying malaria risk [13]. From April 2015 to June 2018, a tripartite China-UK-Tanzania collaboration piloted an adapted version of the “1-3-7” approach, known as the 1,7-malaria Reactive Community-based Testing and Response intervention (1,7-mRCTR), in the high transmission Rufiji district in Tanzania. This pilot study proved highly successful, resulting in an estimated 81% reduction in malaria prevalence from 26% to 4.9% compared with areas that did not receive the intervention [14]. To inform scale-up and consideration for inclusion in the national malaria strategic plan 2021–2025, a trial of the 1,7-mRCTR approach was expanded to additional wards within three high-endemic districts of Tanzania from 2019 to 2021 [15]. This trial included additional control groups and the collection of cost data and evaluation metrics to rigorously evaluate the impact and value of this strategy when deployed at scale.

In this study, a cost-effectiveness analysis of the 1,7-mRCTR approach, as implemented in three high-endemic districts in southeastern Tanzania, was conducted. The costs and cost-savings associated with the introduction of the 1,7-mRCTR approach in the intervention relative to the control wards were estimated. The cost-effectiveness of the intervention was assessed in terms of malaria cases identified, and malaria infections, deaths, and disability-adjusted life years (DALYs) averted.

## Methods

### Population and intervention

The study was conducted in the Rufiji, Kilwa, and Kibiti districts of Tanzania from July 2019–October 2021. Catchment areas of public and private health facilities (*n* = 62) within selected wards in these districts were assigned to an intervention or control group. Treatment assignment was non-random, with assignment decisions made based on malaria incidence rates within each catchment area. A minimum distance of 30 km was imposed to reduce spillover effects across intervention and control groups [15]. The intervention involved targeted community-based testing of villages with high weekly malaria incidence rates (a “campaign”) using malaria rapid diagnostic tests (mRDTs). Subsequent treatment of those testing positive with dihydroartemisinin piperaquine phosphate (DHA-PPQ) followed the national policy guidelines for malaria treatment [16]. Locally trained community health workers conducted the community-level testing and treatment by setting up temporary testing and treatment stations in the high-burden communities. Both control and intervention areas benefited from existing routine malaria interventions (e.g., delivery of long-lasting insecticide treated bed nets, vector control) delivered by the Tanzania National Malaria Control Programme. Additional details on the implementation of the 1,7-mRCTR intervention and the results of the impact evaluation have been published elsewhere [13–15].

Two waves of cross-sectional household surveys were conducted: the first between July and September 2019 and the second between September and October 2021. Households within a given village were selected using a stratified sampling approach. These surveys covered 11,229 households and assessed malaria prevalence at baseline and endline within the intervention and control groups by using mRDTs to test one randomly selected household member from each of three age groups (under 5, ages 5 to 15, and above 15 years), if available to be surveyed. Malaria prevalence was calculated as the total number of positive tests divided by the total number of tests performed. The household surveys also incorporated questions on socio-economic characteristics, including household income and costs associated with malaria illness (e.g., lost productive time and fees paid for healthcare services and treatment). Additional details on household selection methods for surveys and household characteristics at baseline and endline have been published in the impact evaluation of the 1,7-mRCTR intervention [15].

### Costing approach

We assessed costs from a societal perspective, which included the costs of delivering the 1,7-mRCTR intervention, the costs or cost-savings resulting from changes in demand for related health services (i.e., difference in malaria diagnoses made at a health facilities assigned to the intervention vs. control arm over the study period), and the costs or cost-savings experienced by individuals and households due to changes in disease incidence or need for healthcare. We defined health system costs as the 1,7-mRCTR intervention costs plus the costs and cost-savings resulting from changes in provision of routine malaria diagnosis and treatment due to the intervention. Patient costs were defined as the sum of the direct non-medical, productivity, and direct medical costs incurred by patients and their households. Direct non-medical costs represent non-medical expenditures incurred due to illness or to receive care, including food and travel costs incurred while visiting a health facility for malaria testing. Productivity costs represent changes in household economic productivity due to individuals being unwell, or time lost while seeking and/or providing care for sick household members. Direct medical costs represent payments made by patients and their households for medical services and products, including registration and consultation fees, the costs of malaria rapid diagnostic tests, and drug costs [17].

### Cost data collection and analysis

We collected cost data using a combination of micro-costing methods for patient costs and macro-costing methods for programmatic costs [18]. The total programmatic costs required to deliver the 1,7-mRCTR intervention were collected during intervention implementation and apportioned into research-related and intervention-related costs. Research-related costs, which are expenses incurred only for the purposes of the study and not expected under routine implementation, were excluded from the analysis. Patient costs were estimated from household survey data collected at baseline and endline. The productivity component of patient costs was calculated by multiplying lost productive time by the average daily income across all age groups and sites calculated from household survey data.

We assumed that individuals who developed malaria would experience one of three scenarios: they could be diagnosed and treated at a health facility, diagnosed and treated during a 1,7-mRCTR campaign, or remain undiagnosed and untreated for the duration of their illness. The cost of a routine malaria episode diagnosed and treated at a health facility was calculated as the sum of the direct non-medical, productivity, and direct medical costs reported by individuals in household surveys who sought care for malaria symptoms. To calculate the cost of a malaria episode diagnosed and treated during a 1,7-mRCTR campaign, we assumed that an individual testing positive during a 1,7-mRCTR campaign would accrue only one-half of the productivity costs due to illness, as, on average, individuals would be identified halfway through the average duration of illness. We also assumed that individuals diagnosed and treated during a campaign would not experience lost productive time from healthcare seeking as treatment is provided within their community. To calculate the cost borne by an individual with an undiagnosed and untreated episode of uncomplicated malaria, we assumed an individual would lose 3 days of productive time due to illness based on empirical data collected from the household surveys.

All costs collected in Tanzanian shillings were adjusted for inflation to 2022 values using the gross domestic product (GDP) deflator for Tanzania [19]. Costs were then converted to United States Dollars (USD) using the 2022 average market exchange rate of 2329 shillings to 1 USD [20]. Future costs were discounted at 3% [17]. All results are reported in 2022 USD.

### Impact on malaria prevalence and diagnoses

The effect of the 1,7-mRCTR intervention on malaria prevalence was derived from a published impact evaluation that analyzed mRDT positivity from the baseline and endline household surveys [15]. In addition, we used malaria register data from health facilities in intervention and control wards to calculate the total number of passively detected malaria cases over the study period. Data from the 1,7-mRCTR campaigns, including the number of campaigns, tests conducted, and positive mRDTs, were used to calculate the total number of malaria cases diagnosed by reactive case detection in the intervention arm.

### Impact on malaria incidence, deaths, and DALYs

We used OpenMalaria, an open-source transmission-dynamic malaria microsimulation model, to simulate malaria epidemiology within the study region and to assess the impact of the 1,7-mRCTR intervention on health outcomes that could not be assessed empirically: malaria cases, deaths, and DALYs [21]. We initialized the model with a population of 10,000 individuals with age distribution based on the Ifakara district of Tanzania [22]. Using the model, we simulated malaria epidemiology from January 2017 to January 2024 using a five-day time step. Discrete-time population models of mosquitoes followed a seasonally forced pattern using a calibrated parameter value for the average annual pre-intervention entomological inoculation rate (EIR) of 4.8 infectious bites per person-year. A burn-in period of one lifespan established a stable level of immunity within the simulated population. The model was fit to malaria prevalence calculated from the baseline household survey and the effect estimate (4.5 percentage point reduction in malaria prevalence) reported by the impact evaluation [15].

We ran 100 simulations for each of two scenarios: one including the 1,7-mRCTR intervention and one without any intervention to represent the counterfactual (i.e. passive detection only) scenario. Each pair of simulations was initialized using the same random seed. In the intervention scenario, individuals could be treated for malaria through two routes: individuals with symptoms could seek care at a health facility or be screened and treated during a campaign. We utilized monthly deployments of mass screen-and-treat campaigns using mRDTs from September 2019 to September 2021 parameterized from trial data, based on the timing and coverage (i.e. proportion of the population screened) of each campaign. To replicate observed interruptions in campaign deployments from the COVID-19 pandemic and mRDT stock-outs, we modelled no campaign deployments from April–May 2020 and in April 2021.

Model outcomes were aggregated from September 2019 (the date of the first 1,7-mRCTR campaign and baseline prevalence measurement) to January 2024 (28 months after the last campaign, the length of time required for malaria incidence to return to pre-intervention levels) to capture longer-term effects of the intervention on malaria incidence and deaths. These outcomes were also used to evaluate the impact of the intervention on malaria deaths and DALYs. Estimates of years lived with disability (YLDs) per malaria case and years of life lost (YLLs) per malaria death were obtained from the Global Burden of Disease Collaborative Network [23].

### Cost-effectiveness analysis

We conducted a cost-effectiveness analysis using empirical cost data and outcomes from the trial for proximal health effects, and modelled estimates of malaria cases, DALYs, and deaths. Cost-effectiveness results are reported as incremental cost-effectiveness ratios (ICERs), which represent the ratio of incremental costs to incremental health benefits for the 1,7-mRCTR intervention compared with the status quo of passive case detection [24].

We report the results for five cost-effectiveness endpoints: (i) the incremental cost per person treated in a 1,7-mRCTR campaign, (ii) the incremental cost per additional malaria case detected through a combination of passive and reactive case detection, (iii) the incremental cost per incident malaria case averted, (iv) the incremental cost per malaria death averted, and (v) the incremental cost per DALY averted. For the first and second endpoints, incremental costs were defined as the intervention-related programmatic costs. Incremental costs for the third, fourth, and fifth endpoints were defined as the intervention-related programmatic costs plus the cost difference between the intervention and control arms in terms of the costs of malaria diagnoses made at the health facility, costs of diagnoses made in a campaign, and costs associated with undiagnosed and untreated individuals with malaria. This captures the cost savings of reactive case detection through two channels: the reduced burden borne by patients and the health system as individuals are tested and treated in their villages, and lower malaria incidence due to individuals with malaria being identified and treated earlier in the course of illness, which reduces the risk of transmission [25].

### Statistical analysis

We assessed the uncertainty around incremental costs using a non-parametric bootstrap with 10,000 iterations. For incremental effects on malaria incidence, deaths, and DALYs, we used the OpenMalaria simulation results to construct probability distributions describing the additional uncertainty associated with the modelled results. We used a Monte Carlo simulation to combine the different sources of uncertainty, producing a set of 10,000 results representing the overall uncertainty in each outcome of interest. Uncertainty intervals are reported as equal-tailed 95% confidence intervals (*CI*s), scatterplots on the cost-effectiveness plane, and cost-effectiveness acceptability curves [26]. ICERs were compared to willingness-to-pay thresholds based on the effects of changes in health expenditure in Tanzania on survival and morbidity burdens of disease, using an approach described by Ochalek et al. [27].

### Sensitivity analysis

To assess parametric uncertainty, we performed a one-way deterministic sensitivity analysis on key parameters in the analysis, including the intervention-related programmatic cost, duration of illness for undiagnosed and untreated individuals, and the duration of illness before seeking treatment [26]. We varied the intervention-related programmatic cost by ± 15% from the base case and the duration of illness by ± 1.5 days from the base case.

## Results

### Cost results

The average daily income reported in the household survey was 0.9 USD (95% *CI*: 0.7, 1.1). Household survey results showed that 15% of individuals sought care for malaria symptoms on the day of fever onset, 52% within 1 day, 26% within 3 days, and 7% waited over 3 days before seeking care. Based on these results, we assumed that on average, individuals lose 2.5 days of productive time after symptom onset to seek care at a health facility and receive treatment. The direct non-medical cost (including food and travel costs) and productivity costs of a routine malaria episode requiring a visit to the health facility were estimated as 0.9 USD (95% *CI*: 0.9, 1.1) and 2.2 USD (95% *CI*: 2.0, 2.7), respectively. The average direct medical cost, including the costs of medical consultation and care, was estimated as 2.0 USD (95% *CI*: 1.9, 2.3). Summing these three values, the total patient cost of a routine malaria episode diagnosed and treated at a health facility was 5.1 USD (95% *CI*: 4.8, 5.7) (Table 1). The total patient cost of a case diagnosed in a 1,7-mRCTR campaign was estimated as 0.9 USD (95% *CI*: 0.7, 1.1). The total cost of implementing the 1,7-mRCTR intervention (excluding research-related costs) was 1,050,873 USD over the study period, or 5327 USD per 1000 population. Cost drivers for the intervention were personnel costs (46.7%) and supplies (29.0%), followed by training (7.7%) and transport costs (5.7%). The overall incremental costs of the intervention considering the cost-savings from fewer health facility visits for malaria testing and treatment and the consequences of lower malaria incidence were estimated as 3093 USD (95% *CI*: 2750, 3315) per 1000 population.

**Table 1:** Patient costs associated with routine malaria visit to health facility

Cost Category	Mean (95% *CI*)
Direct non-medical costs	
Travel/Transportation	0.8 USD (0.6, 0.9)
Food	0.2 USD (0.1, 0.3)
Total	1 USD (0.9, 1.1)
Productivity costs	
Daily income	0.9 USD (0.7, 1.1)
Total*	2.2 USD (2.0, 2.7)
Direct medical costs	
Registration fee	0.4 USD (0.4, 0.5)
Consultation fee	0.1 USD (0.1, 0.1)
Test in facility	0.3 USD (0.2, 0.3)
Drug cost in facility	0.7 USD (0.5, 0.9)
Drug cost outside facility	0.4 USD (0.4, 0.5)
Total	2.0 USD (1.9, 2.3)
Routine cost of malaria episode at health facility	5.1 USD (4.8, 5.7)

^*^Total productivity costs were calculated by multiplying the average daily income by 2.5 days, the sum of the average duration of fever before seeking care and time spent traveling to the health facility

### Impact on malaria prevalence and diagnoses

Results from the impact evaluation showed that over the intervention period, malaria prevalence declined from 27.4% at baseline to 11.7% in the intervention arm and from 26.0% to 16.0% in the control arm, with the 1,7-mRCTR intervention estimated to have produced a 4.5 (95% *CI*: 2.3, 6.7) percentage point decrease in malaria prevalence [15]. In the control arm, there were 659 malaria diagnoses per 1000 population (*n* = 27,197) at health facilities over the two-year study period. In the intervention arm, 684 individuals per 1000 population (*n* = 134,966) were diagnosed with malaria and treated at health facilities. Over the 1,7-mRCTR campaigns, 244,757 total individuals were tested with mRDTs, of which 82,742 (33.8%) tests were positive, resulting in 420 malaria diagnoses per 1000 population. Overall, the combination of passive and reactive case detection in the intervention arm led to 1104 malaria diagnoses per 1000 population over the study period, an additional 445 cases per 1000 diagnosed compared to passive detection alone.

### Impact on malaria incidence, deaths, and DALYs

Based on the simulation modelling results, we estimated that the 1,7-mRCTR intervention averted 427 (95% *CI*: 218, 637) incident malaria cases per 1000 population and 0.2 (95% *CI*: 0.0, 0.8) malaria deaths per 1000 population, relative to the control arm. For every percentage point decline in malaria prevalence the intervention averted an average of 95.2 incident cases (95% *CI*: 91.8, 98.3) and 0.0 (95% *CI*: 0.0, 0.2) deaths per 1000 population. We estimated that on average, the 1,7-mRCTR intervention averted 19.1 (95% *CI*: 4.3, 74.0) DALYs per 1000 population.

### Cost-effectiveness of 1,7-mRCTR

The average cost per person tested in a campaign was estimated as 4.3 USD and the average cost per positive test in a campaign was 12.7 USD. For overall malaria diagnoses within the study area (considering cases detected through both campaigns and routine healthcare), the incremental cost per additional case detected was 12.0 USD.

**Fig. 1:**
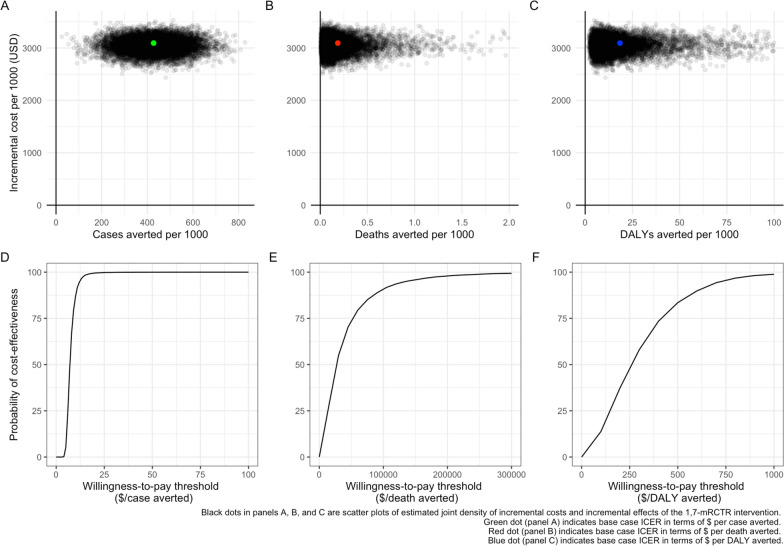
Cost-effectiveness of the 1,7-mRCTR intervention. Panel **A** incremental costs per malaria case averted. Panel **B** incremental costs per malaria death averted. Panel **C** incremental costs per DALY averted. Panel **D** cost-effectiveness acceptability curve for the cost per malaria case averted. Panel **E** cost-effectiveness acceptability curve for the cost per malaria death averted. Panel **F** cost-effectiveness acceptability curve for the cost per DALY averted. * Cost-effectiveness acceptability curves show the probability that the 1,7-mRCTR intervention is cost-effective at different willingness-to pay thresholds. $ means USD

Compared to passive malaria detection, the ICERs for the 1,7-mRCTR intervention were 7.3 USD (95% *CI*: 4.7, 14.0) per incident malaria case averted, 16,884 USD (95% *CI*: 3757, 185,604) per malaria death averted and 163 USD (95% *CI*: 41, 700) per DALY averted. Using a willingness-to-pay threshold of 417 USD per DALY averted, the 1,7-mRCTR intervention was found to be cost-effective in this setting, with a probability of 82% (Fig. 1).

### Sensitivity analysis

**Fig. 2:**
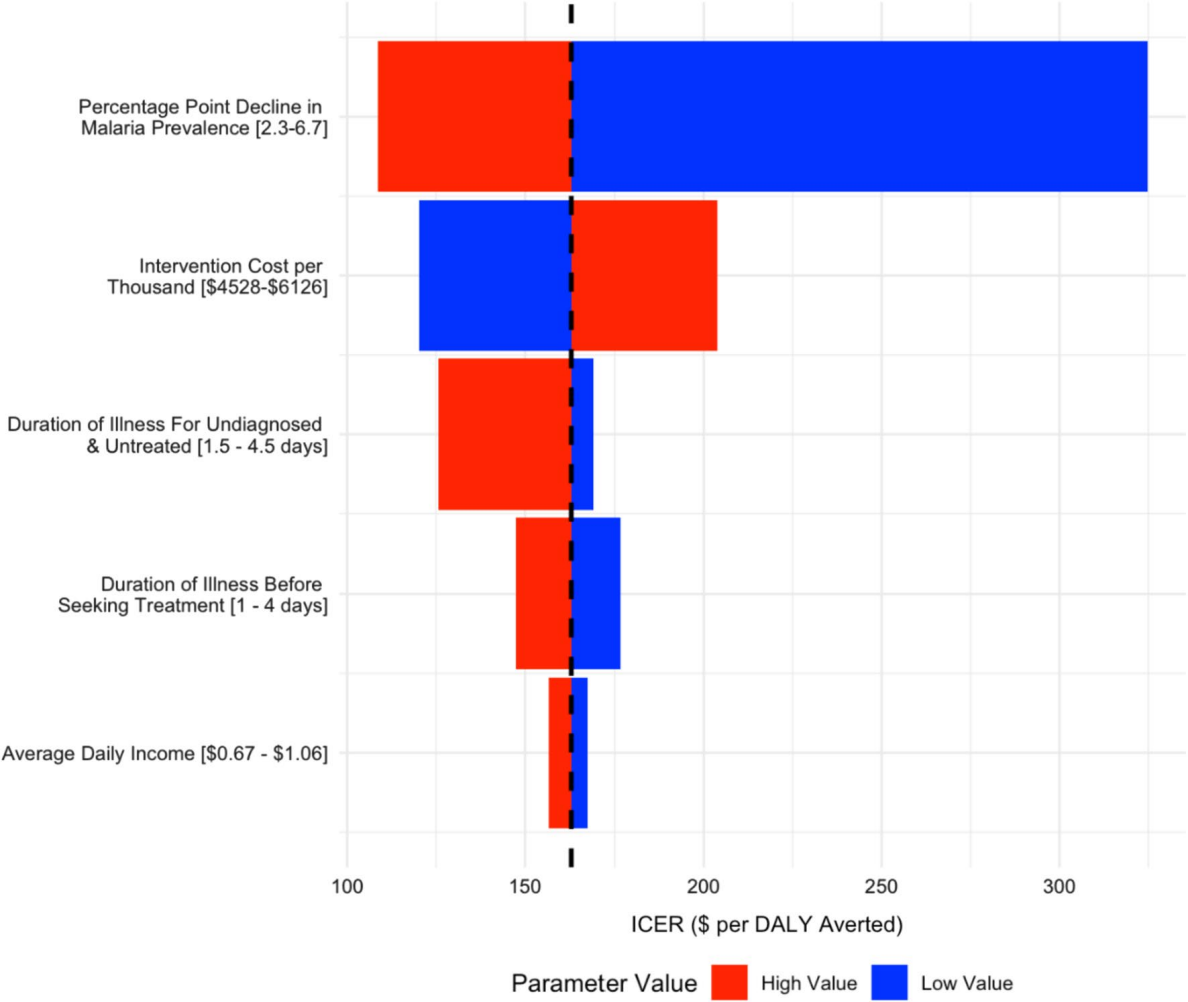
Deterministic one-way sensitivity analysis on key parameters illustrating the impact of parameter value on the incremental cost-effectiveness ratio (ICER) in terms of USD per disability-adjusted life year (DALY) averted. The dashed black line indicates the base case ICER of 163 USD per DALY averted. Parameters and value range assessed from low to high are indicated on the y-axis, while the corresponding ICER is indicated on the x-axis, using red for the ICER associated with the high parameter value and blue for the ICER associated with the low parameter value. $ means USD

We conducted a deterministic one-way sensitivity analysis to understand how model parameters affected the incremental cost per DALY averted (Fig. 2). These results showed that the effect estimate of the 1,7-mRCTR intervention on malaria prevalence (base case of 4.5 percentage point decline in malaria prevalence) was the most influential parameter. When this input was varied from a 2.3 percentage point decline in malaria prevalence to a 6.7 percentage point decline, the ICER decreased from 325 USD per DALY averted to 109 USD per DALY averted. Other influential parameters were intervention-related programmatic costs, duration of illness for undiagnosed and untreated individuals with malaria, duration of illness for those who sought treatment, and average daily income.

## Discussion

In this study, we conducted an economic evaluation alongside a trial of the 1,7-mRCTR intervention in rural Tanzania, a setting with moderate-to-high malaria transmission. Using trial data on costs and immediate health outcomes, and a mathematical model to simulate the long-term impact on malaria incidence, deaths, and DALYs, we found that the 1,7-mRCTR intervention had an incremental cost-effectiveness ratio of 163 USD per DALY averted. This cost-effectiveness ratio falls below the willingness-to-pay threshold of 417 USD per DALY averted, indicating that the strategy would be cost-effective in this setting.

To our knowledge, no prior studies have reported the cost-effectiveness of modified RACD strategies such as 1,7-mRCTR for malaria control in moderate-to-high transmission settings. One study assessing the cost-effectiveness of RACD relative to other case-finding strategies for malaria has been conducted in the low-transmission setting of Namibia [28]. When compared to reactive focal mass drug administration and reactive focal vector control strategies, ICERs for RACD were 117–955 USD per DALY averted (adjusted from 97 to 794 in 2017 USD to 2022 USD, using the US Bureau of Labor Statistics Consumer Price Index Inflation Calculator) [28, 29]. Mass screen and treat (MSAT) interventions (which do not require the surveillance and focal response component of RACD strategies), have been found to be cost-effective in moderate to high transmission settings [30]. In southern Zambia, a moderate malaria transmission setting, the ICER for MSAT from a provider perspective was estimated to be 1150 USD per DALY averted (adjusted from 894 in 2013 USD to 2022 USD) [30]. This is seven times higher than our base case ICER of 163 USD per DALY averted for the 1,7-mRCTR intervention, although our estimate includes patient costs and cost-savings.

Our analysis suggests that modified RACD strategies such as 1,7-mRCTR, while resource-intensive, may be a cost-effective approach for reducing malaria burden in high-transmission settings, especially when compared to mass deployment of antimalarial drugs or testing and treatment. This study benefited from the use of trial data to obtain patient costs associated with malaria illness, diagnosis, and treatment, as well as programmatic data on the implementation costs of the 1,7-mRCTR intervention. Furthermore, by using a locally calibrated simulation model, we were able to assess the long-term health effects of the 1,7-mRCTR intervention, allowing us to report cost-effectiveness in terms of malaria deaths and DALYs averted, and include the costs associated with undiagnosed and untreated malaria in the cost analysis.

This study had several limitations. First, the 1,7-mRCTR intervention was implemented alongside other routine malaria control programs in both intervention and control areas. Of note, larviciding was conducted alongside the 1,7-mRCTR intervention in Rufiji District alone, which would have an additional impact on parasite prevalence and therefore malaria prevalence. Results from the impact evaluation of the 1,7-mRCTR intervention show that the effect of the intervention on malaria prevalence was largest in the Rufiji district. The Rufiji district also had the lowest baseline malaria prevalence of all three districts, suggesting that the intervention is most effective in moderate transmission settings and alongside larviciding efforts. As we were unable to isolate the programmatic cost by district, the costs of larviciding, or the effect of the 1,7-mRCTR intervention on malaria prevalence alone, we included the costs of larviciding in the intervention arm in this cost-effectiveness analysis. Second, we needed to make several assumptions about the duration of illness for individuals who developed malaria and used average daily income from the household surveys to calculate productivity loss averaged across all ages and sites. We addressed these concerns by conducting a sensitivity analysis on the duration of illness and average daily income. Third, our estimates of malaria incidence, deaths, and DALYs averted relied on a mathematical model fit to the empirical endpoints of the study. While reporting these long-term health outcomes allows the intervention to be compared with a wider range of interventions, this modelling step adds additional uncertainty to the study outcomes. Finally, the study was conducted in three districts in Tanzania. While this may be representative of a rural, high malaria transmission setting, additional studies are needed to understand how intervention costs and effectiveness vary across settings, and how interventions such as 1,7-mRCTR can optimally be incorporated into regional and national malaria control programs for countries like Tanzania.

## Conclusions

The 1,7-mRCTR intervention aims to reduce malaria prevalence in moderate-to-highmoderate-to-high transmission areas by utilizing existing healthcare infrastructure and a locally tailored, community-based malaria response. This strategy involves the integration of primary healthcare services, community outreach, and focused resource utilization to enhance access to healthcare in rural settings. We found the 1,7-mRCTR intervention to be impactful and cost-effective in a rural, moderate-to-high transmission region of Tanzania. This comprehensive model holds promise for efficiently addressing the challenge of malaria in high-endemic settings.

## Data Availability

The datasets used and/or analyzed during the current study are available from the corresponding author on reasonable request.

## Abbreviations

1,7-mRCTR: 1,7-Malaria Reactive Community-based Testing and Response intervention
CCDC: Chinese centers for disease control
COVID-19: Coronavirus disease 2019
DALY: Disability-adjusted life year
EIR: Entomological inoculation rate
ICER: Incremental cost-effectiveness ratio
RACD: Reactive case detection
mRDT: Malaria rapid diagnostic test
WHO: World Health Organization
YLDs: Years lived with disability
YLLs: Years of life lost due to disease
